# Costs and savings associated with a pharmacists prescribing for minor ailments program in Saskatchewan

**DOI:** 10.1186/s12962-017-0066-7

**Published:** 2017-04-11

**Authors:** Ellen Rafferty, Mohsen Yaghoubi, Jeff Taylor, Marwa Farag

**Affiliations:** 1 School of Public Health, University of Saskatchewan, 104 Clinic Place, Room 3334, Saskatoon, SK S7N 5E3 Canada; 2 College of Pharmacy and Nutrition, University of Saskatchewan, Saskatoon, Canada

**Keywords:** Pharmacists prescribing, Minor ailments, Cost analysis, Economic impact, Societal and public payer perspectives, Cost savings, ROI

## Abstract

**Background:**

Health care systems around the world have started to develop pharmacists prescribing for minor ailments (PPMA) programs. These programs aim to improve the efficiency of care, reduce physician visits, and increase the accessibility to prescription medication (Rx). This study performed an economic impact analysis of the pharmacists prescribing for minor ailments program in Saskatchewan.

**Methods:**

We measured costs for the program and the alternative scenario (i.e. no PPMA program) from a public payer and societal perspective, using primary data on pharmacists prescribing consultations in Saskatchewan. Furthermore, we calculated public payer and societal savings, and return on investment ratios for the program, as well as projecting the costs and benefits over the next 5 years.

**Results:**

Overall, we found that from a societal perspective, the Saskatchewan PPMA program saved the province approximately $546,832 in 2014, while according to the public payer perspective, the program was only marginally cost-saving in 2014. After 5 years of implementation, from a societal perspective, cumulative cost savings were projected to be $3,482,660, and the return on investment ratio was estimated to be 2.53.

**Conclusions:**

Our results demonstrate that this type of program may prove cost-saving and lead to improved access to the health care system in Canada, especially if savings to society are considered. This type of PPMA program may prove economically feasible and beneficial in many countries considering expanding pharmacists scope of practice.

**Electronic supplementary material:**

The online version of this article (doi:10.1186/s12962-017-0066-7) contains supplementary material, which is available to authorized users.

## Background

Health care systems around the world have started to develop pharmacists prescribing programs with the aim to improve the efficiency of care, reduce physician visits, and increase the accessibility to prescription medication [1, 2]. Although there is a range of models for increasing the scope of practice of pharmacists, one area of interest is in pharmacists prescribing for minor ailments (PPMA) (i.e. self-limiting and self-diagnosed health problems). In one study in Scotland, minor ailments, defined in the study as health problems suitable for management by pharmacists, currently account for 13.2% of visits to general practitioners and 5.3% of visits to the emergency room (ER) [3]. The same study estimated that minor ailments conceivably cost the Scottish health care system £1.1 billion USD ($1.9 billion CAN) in resources per year.

Scotland and Northern Ireland were some of the first countries to implement a pharmacists prescribing program, with pharmacists being able to prescribe independently in some areas since 2006 [4]. Currently all provinces in Canada have some level of pharmacists prescribing, however there is a large disparity in terms of the authority and scope within which pharmacists can prescribe [1]. Most provinces have pharmacists prescribing program specific to minor ailments in place.

In Saskatchewan, beginning March 2011, pharmacists were able to prescribe for a variety of minor ailments from a list of medications previously available only from a physician. At present, pharmacists can consult and prescribe for 17 minor ailments including, acne, cold sores, allergic rhinitis, oral aphthous ulcer, oral thrush, diaper dermatitis, atopic dermatitis, dysmenorrhea, gastroesophageal reflux disease (GERD), headaches, hemorrhoids, musculoskeletal pain, skin infections (impetigo and folliculitis) and tinea skin infections (athlete’s foot, jock itch, and ringworm). Saskatchewan currently pays $18 per pharmacist consultation for pharmacists to prescribe for these ailments, the first provincial government to cover such costs. Part of the motivation for starting the Saskatchewan PPMA program (and similar programs), was to decrease costs to the health care system while increasing accessibility and patient satisfaction [5].

Research shows that pharmacists prescribing programs may allow general practitioners (GPs) to focus on more complex cases. Currently, between 10 and 20% of GPs workload and 5% of ER consultations in some countries are for minor ailments [3]. Changing GP workload and case mix is important in Canada and many developed countries, where wait times to see a GP can be over 3 weeks [6]. In Scotland, following the implementation of the Minor Ailments Service, where community pharmacists could prescribe for 12 conditions, there was a 38% transfer from GP to pharmacists prescribing for those conditions [7]. A similar study in England showed that while overall number of GP visits did not decrease following the expansion of a pharmacists prescribing program for minor ailments, they did see a reduction in the proportion of visits that were for minor ailments [8]. Therefore, even if the PPMA program broke even in terms of costs and savings, it may help the system run more efficiently and therefore increase the ability of the health care system to treat patients in a timely manner, as well as improve patient satisfaction. However, the specific impacts of pharmacists prescribing on GP workload and case mix has yet to be shown in Canada, and will be an important area of research in the future.

PPMA programs are not the only alternative to GP visits for minor ailments. One common option is patients’ self-care. For instance, the European Union set up a pilot project to examine and support self-care initiatives in the EU [9]. Self-care is a broader concept that encompasses a wide set of interventions and initiatives; they include information-based initiatives such as media campaigns to increase health literacy and action-based initiatives such as legal changes that change the scope of practice of health practitioners allowing pharmacists to prescribe medications for example [9]. A study of self-care by Willemsen and Harrington [10] found that even if 16% of people who saw a physician for their mild cold or flu symptoms used over the counter (OTC) medication or self-care instead, it would save the Canadian government $98 million per year (or alternatively free up resources to provide greater access to a physician for half a million Canadians). However, evidence shows that while OTC medications can be effective for certain illnesses, many minor ailments often require prescription medications for effective and long-term treatment. In fact, the Saskatchewan Minor Conditions Survey and the literature demonstrates that a large proportion of individuals who used a pharmacists prescribing program would have seen a GP if the PPMA program was not in place [5, 11]. Furthermore, Willemsen and Harrington [10] concluded that one reason Canadians may choose to consult with a doctor rather than self-treat is due to an inability to find an OTC medication that works for their minor ailment.

A recent systematic literature review of 2010–2015 economic evaluations of pharmacy services in hospital and community-based settings in publicly funded health systems worldwide found that these pharmacy services provided clinical benefits which include improved patient outcomes and reduction in adverse use of medications [12]. The findings of the review also indicated that community pharmacy services were considered cost-effective in 8 out of 10 studies [12]. The review highlighted the importance of economic evaluations to provide input to policy makers to help direct resources to their best use [12]. With significant variations in programmatic goals and evaluation, pharmacist training, and reimbursement rates across provinces and countries, and as more regions develop these novel programs, it is important they begin to learn from each other. Most evaluations of pharmacists prescribing programs have studied patient, pharmacist and GP satisfaction and their acceptance of the program, as well as pharmacist consultation skills [13]. To our knowledge, there have only been a few reports that have evaluated the costs and savings related to a pharmacy prescribing program in particular [14, 15].

Moreover, much of the empirical and evaluative work has focused on the UK, and very few analyses from other countries are available to offer a comparative viewpoint [13]. No study has analysed the effectiveness or costs and savings from current pharmacists prescribing programs in Canada [13]. More of these economic studies are needed to assess the cost effectiveness of these programs. Moreover, further knowledge of the costs and savings associated with these novel prescribing programs could help countries make decisions on whether to implement their own PPMA programs.

The objectives of this paper are to perform an economic impact analysis of the pharmacists prescribing for minor ailments program in Saskatchewan, including (1) the current costs and savings of the program from both the public payer and societal perspective and (2) the costs and savings of the program for a time horizon of five years after implementation.

## Methods

This study performed an economic impact analysis of the Pharmacists Prescribing for Minor Ailments Program (PPMA) in Saskatchewan using primary data on pharmacists prescribing consultations recorded since program inception and the Saskatchewan Minor Conditions Survey, an online questionnaire administered to individuals who had consulted a pharmacist as part of the PPMA in Saskatchewan [5]. Furthermore, we used the Saskatchewan physician schedule of benefits and the Canadian Institute for Health Information’s (CIHI) discharge abstract database, as well as secondary data from published materials. Using these data sources we were able to calculate the costs and savings when the Saskatchewan PPMA program was in place and compare them to the situation in the absence of PPMA program (alternative scenario). We measured costs for the program and the alternative scenario (i.e. no PPMA program) from both a public payer (i.e. costs and savings to the health care system) and societal perspectives (i.e. costs and savings to the health care system, the patient and society). Since in the health care market, benefits are frequently gained from cost avoidance rather than from revenue generating activities, we estimated cost savings. Therefore, for the purposes of our analysis, benefits were considered to be the potential monetary savings of using the PPMA program in comparison to the alternative. We also calculated the return on investment (ROI) from both the public payer and societal perspectives. Return on investment (ROI) has been used as a measure to quantify the value of a heath care program within the health care system in important economic evaluations in the past [16–19]. The advantage of presenting a ROI ratio is that it provides a metric for evaluating the financial consequences of investments in future.

Finally, using the societal perspective, we estimated a projection of costs and benefits over five years. We present a detailed description of the methods in the following sections. For a full list of parameters and the data sources see Table 1.

**Table 1:** Parameters table

Item (base case)	Base estimate	Reference
Pharmacy prescribing		
Number of pharmacy consultations in 2014	10,739	SK health department consultation data
Pharmacists minor ailment consultation fee (Can$)	18	SK health department consultation data
Average cost per prescription for minor ailment (Can$)	30.20^a^	SK health department consultation data
Prescription payments by the Saskatchewan (SK) government (%)	0.46	National health expenditure database 2013
Average cost of OTCs (Can$)	12	Expert opinion
Number of pharmacists trained	1484	CPDPP^b^
Training costs per pharmacists (Can$)	210	CPDPP
Training costs per pharmacists (Can$)-online	156.7	CPDPP
Annual training cost (Can$)	61,212	Calculated
The wait-time and duration of a pharmacy consultation (hour)	0.28	[24]
Average distance to pharmacy (km)	1.6	[39]
Cost per km drive (Can$)	0.123	Calculated
The value of an individual’s time per hour(Can$)	24.96	Statistics Canada [25]
The value of an individual’s time per working day (Can$)	199.68	Statistics Canada [25]
Alternative scenarios		
PPMA program users who would have gone to bought OTC	43%	SK minor conditions survey
PPMA program users who would have gone to the GP	35%	SK minor conditions survey
PPMA program users who would have gone to the ER	03%	SK minor conditions survey
Cost of GP visit (Can$)	66.4	[22]
Cost of ER visit (Can$)	138	[23]
The wait time and duration of a GP consultation (hour)	1.75	Expert opinion
The wait time and duration of a ER visit (hour)	4.6	[38]
Average distance to GP (km)	3.2	[39]
Average distance to ER (km)	24.8	[39]

*Markup fee* a pharmacy mark-up refers to any additional amount a pharmacist may charge for a drug, above the original drug cost. The mark-up is applied to help pay for the costs of running the pharmacy, this fee calculated based on percentage of drug price which varies between 10 and 30% as following: 30% for drug cost up to $6.30; 15% for drug cost between $6.31 and $15.80; 10% for drug cost of $15.81 to $200.00, and a maximum mark-up of $20.00 for drug cost over $200.00 [58]

*Dispensing fees* this fee covers services such as: talking about your treatment with you, maintaining and checking your medication records and providing drug information to your doctors. The maximum dispensing fee is $11.40 (effective September 1, 2015) [59]

^a^Average cost per prescription = Average price per medication + Markup per medication (%) + dispensing fees ($11.40)

^b^Continuing professional development for pharmacy professionals

### Public payer perspective

#### Costs of the PPMA program

Using a public payer perspective, we considered only direct costs to the health care system associated with the PPMA, including pharmacist remuneration for service delivery based on the current pharmacist remuneration fee of $18 per consultation and the cost of publically funded prescriptions. Using consultation records kept by the health department from 2014, we estimated there were 10,739 pharmacy consultations for minor ailments per year in Saskatchewan.

Prescription drugs used for each minor ailment and their corresponding drug acquisition costs were obtained from Drug Plan and Extended Benefits Branch of Saskatchewan Health [20]. “According to the agreement between Saskatchewan Health and pharmacy proprietors, the prescription cost is calculated by adding the acquisition cost of the drug material, the submitted mark-up and dispensing fee (up to a maximum)” (Government of Saskatchewan Extended Benefits and Drug Plan) [18]. Therefore, we calculated total prescription drug price for each drug by adding an average mark-up of 10–30% of the drug acquisition cost following the rules of the Government of Saskatchewan and the current Saskatchewan dispensing fee of $11.00. We then calculated the average prescription drug price for each minor ailment and for all minor ailments included under the scope of the program. The average prescription drug price for minor ailments prescription was estimated to be $30.20. (Additional file 1).

For recording of transactions by the Saskatchewan health department and subsequent remuneration, the minor ailment consultation must end in a prescription. Therefore we assumed all consultations reported ended in a prescription. Subsequently, to calculate the cost of prescription to the health care system each year, we multiplied the average cost per prescription by the total number of consultations per year (10,739) and by 46%, which represented the percentage of prescription costs paid by the government of Saskatchewan in 2013 (National Health Expenditure Database 2013; Canadian Institute for Health Information) [21]. We calculated total program delivery costs from the public payer perspective to be $193,302.

#### Costs of the alternative

The Saskatchewan Minor Conditions Survey asked patients who had used the PPMA program “If you had not asked for help, what would you have done instead?”. Using these results we determined where patients with minor ailments would have sought care if the PPMA program was not available. The survey concluded that 35% of patients would have seen a GP, 3% would have gone to the ER, 43% would have self-treated with OTC medication and 14% would have done nothing or used something at home [5]. These estimates were slightly more conservative than a previous survey by Westerlund et al. [11], which suggested that up to 56.8% of individual who obtained a pharmacist prescription would have consulted a GP if the pharmacist program was not in place. The Saskatchewan schedule of benefits provided the cost of an exploratory GP appointment ($66.40) [22]. Furthermore, CIHI’s Discharge Abstract Database estimated the average cost of an emergency room visit is Saskatchewan at $138 per case [23]. Therefore, the total cost of GP and ER visits in the alternative scenario (cost of an appointment*number of appointments) was estimated to be $249,574 and $44,459, respectively. Finally, to estimate cost of prescriptions in this scenario, we multiplied the average cost per prescription ($30.20) by the total number of GP and ER visits per year and the percentage of prescription costs paid by the government (46%), and concluded prescription in this scenario would total $56,681 per year. There were no costs from the public payer perspective associated with individuals who did nothing, used something at home or self-treated with OTC medication.

### Societal perspective

#### Costs PPMA program

To calculate the costs of running the PPMA program under the societal perspective, we included both the direct health care costs outlined above and the indirect costs to society and the individual patient costs (i.e. travelling and waiting time, lost productivity, private prescription costs, over-the-counter medications, and training cost of pharmacists). The total lost time for a pharmacy consultation was assumed to be 2 min waiting time and 15 min duration of consultation [24]. Therefore, to calculate opportunity cost we multiplied the lost time at the pharmacy by the number of pharmacy minor ailment consultations in 2014 and the average wage per hour in Canada ($24.96) (Statistics Canada 2016) [25]. Cost of travelling to a pharmacy was calculated using the average time a spent travelling to a pharmacist obtained from Geographic Accessibility of Community Pharmacies in Canada [26] (approximately 1.6 km), as well as the mean fuel costs per km in Canada ($0.12/km) [27]. Furthermore, for each pharmacy consultation we added the cost per prescribed item paid privately (i.e. out-of-pocket by the individual or paid through private insurance), which we assumed to be 54% (34.5% insurance and 19.5% patient) of total drug costs (National Health Expenditure Database 2013). To calculate training cost of PPMA program, we considered the total number of pharmacists that took the live and online training for minor ailments. Cost of the training for pharmacists for the live sessions was $210.00 and for the online version it was $157.50. All pharmacists in Saskatchewan were trained at the beginning stages of implementation of the PPMA program and individual pharmacists or their employers paid for the cost of training and therefore training costs are only included in the societal perspective. Finally, we capitalized training cost and used the equivalent annual cost (EAC) approach to estimate annual cost of training.

#### Cost of the alternative

In order to calculate societal costs for the alternative scenario, we estimated the direct costs of the alternative, along with the average cost of OTC medications, as well as indirect costs for travel to and wait time for GP visits or the emergency room and productivity loss from inadequate care for minor ailments. First, using expert opinion and a survey of the average cost of OTCs for the five most common minor ailments covered in the PPMA program, we estimated OTC costs at $12 per medication. Cost per OTC medication along with the total number of patients who would have used OTCs in the absence of the PPMA program, projected the total cost of OTCs at $55,413 per year. Moreover, we were able to estimate the productivity loss as a result of the potential lower effectiveness of OTCs as compared to prescription drugs. For this analysis we considered five pharmacist-consulted minor ailments (allergic rhinitis, GERD, headache, cold sores and musculoskeletal pain), which accounted for 63% of pharmacist consultations in Saskatchewan and identified as being minor ailments quite likely to cause time off work and may require prescription medication for resolution. Therefore, we only considered there to be productivity losses for the 57% of individuals who would have self-treated or treated with OTCs as part of the alternative, as everyone in the PPMA program received a prescription. First, we extracted the number of days absent per year for each minor ailment from the literature [28–30]. Second, we estimated total productivity loss per day absent based on the average wage in Canada [25]. Third, we obtained data from clinical trials and a meta-analysis to estimate the relative efficacy of each of the most common OTC (OTC drug versus placebo) and prescription medications (prescription versus placebo) for the five minor ailments [31–37]. Then we estimated incremental efficacy by subtracting efficacy of prescription compared to placebo from the efficacy of OTC compared to placebo. We then calculated the incremental productivity loss cost as a result of using an OTC instead of a prescription drug by multiplying the total estimated productivity loss for each ailment by the incremental efficacy, then calculated total productivity loss for each ailment by multiplying the incremental productivity loss cost for an OTC drug per case by the number who would have used OTC medications for each of the five minor ailments mentioned above in the alternative scenario. Finally, the detailed description of how we estimated the total productivity loss cost for these five minor ailments is presented in Table 2.

**Table 2:** Estimate productivity loss cost associated with OTC drug for five minor ailments

Ailment	Absent days per year	Productivity loss cost^a^	Efficacy (Rx vs. placebo)	Efficacy (OTC vs. placebo)	Incremental efficacy^b^	Incremental productivity loss cost for OTC^c^	Number of OTC requests in 2014	Total productivity loss cost for OTC^d^
Allergic Rhinitis	3.6 [28]	$718.8	0.59 [31]	0.21 [31]	0.38	$273.2	660	$180,301
GERD	5.4 [29]	$1078.3	0.45 [32]	0.36 [32]	0.09	$97.0	135	$13,186
Headache	3.8 [29]	$766.8	0.51 [33]	0.41 [34]	0.1	$76.7	80	$6,165
Cold sore	2.7 [30]	$543.1	0.38 [35]	0.08 [35]	0.3	$162.9	1914	$311,924
Musculoskeletal	7.2 [29]	$1437.7	0.48 [36]	0.4 [37]	0.08	$115.0	121	$13,996

^a^Productivity loss cost = The value of an individual’s time per working day ($199.68)* absent day per year

^b^Incremental efficacy = Efficacy (Rx vs. placebo) − efficacy (OTC vs. placebo)

^c^Incremental productivity loss cost for OTC = Productivity loss cost* incremental efficacy

^d^Total productivity loss cost for OTC = Incremental productivity loss cost for OTC* number OF OTC request

Furthermore, we estimated the total time a patient spends at a family physician or walk-in clinic for a minor ailment would be 105 min (90 min wait time and 15 min per consultation). These estimates were based on data obtained through consultations with walk-in clinics in Saskatoon. Moreover, we estimated that 4.6 h was the average time a patient waits for care at emergency department units in Canada [38]. To calculate productivity lost due to time spent waiting for care, we multiplied the average length of time spent for GP consultations and ER visits by the number of GP appointments and ER visits in the alternative scenario and the value of an individual’s time (i.e. average wage).

Finally, we obtained the average length of time a person spends travelling to the GP clinic, ER department and pharmacy for OTC medications, from the Geographic Distribution of Physicians in Canada [39], which showed an average distance to a GP clinic, ER and pharmacy of 3.2, 24.8 and 1.6 km, respectively. Using the same travel costs per km of $0.12, we estimated the total travel cost in the alternative scenario to be $3370 per year. Finally, we include the cost of prescriptions from GPs and ERs that were paid privately (i.e. private insurance or out-of-pocket).

### Ratio of costs to savings

We calculated the total costs and savings of running the PPMA program in 2014 from both the public payer (direct health service costs) and societal (direct and indirect costs) perspective by taking the difference between total costs of the PPMA program and the total costs of the alternative scenario (i.e. no pharmacist prescribing).

We calculated the return on investment ROI ratio of the PPMA using as follows:

$${\text{ROI}} = {\text{Total net cost savings}}\, {\text{(Total cost-savings}}- {\text{investment cost)}}/ {\text{investment costs}}$$

In this formula, we assumed the initial investment costs were the number of pharmacy consultations multiplied by the consultation fee under the public pay perspective; and these costs in addition to pharmacist training costs under the societal perspective.

### Projection of costs and benefits over the next five years

In order to establish the future benefits and costs of the pharmacy prescribing program, we extended the incremental annual direct and indirect costs and benefits of the PPMA program over 2015–2019. Using data on the number of pharmacist consultations in Saskatchewan for 2013, 2014 and 2015, we found that the number of pharmacist consultations has been increasing over these years but at a decreasing rate. The increase in the total number of consultations from 2013 to 2014 was 45% and from 2014 to 2015 was 20%. Therefore, we assumed that the number of pharmacist consultations would continue to increase but that the rate of increase will continue to decrease over the years. We assumed the increase rate would be 8.9, 4, 1.8 and 0.08% in the next four year (2016–2019). We held all other parameters constant, however we discounted any future costs and benefits based on the CADTH recommendations of 5%. This analysis allowed us to estimate a cumulative present value of cost and benefit over 5 years (Table 3).

### Scenario and sensitivity analysis

To account for the uncertainty in parameter estimates, we conducted one-way sensitivity analysis, where we varied one parameter at a time to see the impact on the overall results. We choose to vary five parameters (cost per prescription, cost of OTCs, wait times for GP and pharmacy, and % prescriptions GP/ER) based on their likely impact on the results and the level of uncertainty in how we derived the estimate. Moreover, using scenario analysis we calculated the worst and best possible return on investment for costs and savings from the PPMA program, both for the current year and the projection over the next 5 years. See Table 4 for a complete description of the parameters and values used in the sensitivity analysis.

## Results

**Fig. 1:**
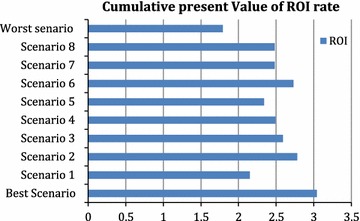
Sensitivity analysis: cumulative present value of return on investment (ROI) according to different scenarios

Overall, we found that PPMA program saved Saskatchewan approximately $801,347 and $201,552 in 2014 from societal and public payer perspectives, respectively. However, the public payer perspective was only marginally cost-saving in 2014, averting $8250 in public costs (Table 3). Our results demonstrated that the PPMA program would increase the total costs averted every year, even assuming a decreasing rate of the increase in the number of pharmacist consultations per year. After 5 years, we estimated that the cost savings would be $3,482,606 and $47,385 from societal and public payer perspectives respectively. Also cost-savings per consultation would be $48.73 according to the societal perspective. Finally, return on investment ROI ratio from the societal perspective will be 2.53 and 0.04 from public payer perspective (Table 3). Sensitivity analysis demonstrated uncertainty in average cost per prescription for minor ailments had the biggest impact on our results, with the return on investment ranging between 2.15 and 2.78, depending on the cost of the prescription. However, from a societal perspective, the results from all the sensitivity analyses we performed were positive. In the best case scenario, the ROI in the next five years increased by 20.16%. Conversely, in the worst case scenario the ROI decreased by 29.25% (Table 4, Fig. 1).

**Table 3:** Costs of the PPMA program and the alternative from the public payer and societal perspectives in 2014 and in the next 5 years

	Public payer perspective	Societal perspective	Public payer perspective	Societal perspective
	2014		2015–2019	
*PPMA program*				
Investment cost of PPMA^a^	$193,302	$254,514	$1,110,134	$1,375,154
Running cost of PPMA	$149,162	$401,432	$856,641	$2,305,430
*Alternative scenario*				
Cost of alternative	$350,715	$1,202,780	$2,014,161	$7,163,244
*Benefits of PPMA*				
Benefit (cost-saving)^b^	$201,552	$801,347	$1,157,519	$4,857,814
Net cost–saving^c^	$8,250	$546,832	$47,385	$3,482,660
Cost–saving per consultation^d^	$0.77	$50.92	$0.66	$48.73
ROI^e^	0.04	2.15	0.04	2.53

^a^Investment cost of PPMA = (Total number of consultation* fee) under public payer perspective

Investment cost of PPMA = (Total number of consultation* fee) + annual training cost under societal perspective

^b^Benefit (cost-saving) = (Cost of alternative − cost of PPMA)

^c^Net cost-Saving = (Cost-saving − investment cost of PPMA)

^d^Net cost–saving per consultation: (Net cost saving/total number of consultation)

^e^ *ROI* net cost–saving/investment cost of PPMA

**Table 4:** Sensitivity analysis base, low and high estimates and results

Cumulative present value in scenario analysis (societal perspective)						
Scenario	Description	Cost of PPMA^a^	Cost of alternative	Net cost–saving	ROI	% Change ROI^b^
Base	Base scenario	$3,680,584	$7,163,244	$3,482,660	2.53	–
Scenario 1	Average cost per prescription for minor ailment (high)-$43.64	$4,528,280	$7,485,368	$2,957,088	2.15	−15.02%
Scenario 2	Average cost per prescription for minor ailment (low)-$21.22	$3,127,044	$6,952,899	$3,825,854	2.78	9.88%
Scenario 3	Average cost of OTCs (high)-$15	$3,680,584	$7,242,804	$3,562,219	2.59	2.37%
Scenario 4	Average cost of OTCs (low)-$12	$3,680,584	$7,110,204	$3,429,620	2.49	−1.58%
Scenario 5	Wait time and duration of a GP consultation—low (1.25)	$3,680,584	$6,893,198	$3,213,267	2.34	−7.51%
Scenario 6	Wait time and duration of a GP consultation—high (2.25)	$3,680,584	$7,423,637	$3,752,052	2.73	7.91%
Scenario 7	Wait time at Pharmacy (5 min)	$3,757,553	$7,163,244	$4,780,844	2.48	−1.98%
Scenario 8	GP and ER prescribe medication in 90% of cases	$3,680,584	$7,092,478	$3,411,893	2.48	−1.98%
Best	Low Ave cost per prescription/high Ave cost of OTC/high wait time and duration of GP consultation	$3,127,044	$7,301,851	$4,174,807	3.04	20.16%
Worst	High Ave cost per prescription/low Ave cost of OTC/low wait time and duration of GP consultation/High wait time at pharmacy/GP and ER prescribe medication in 90% of cases	$4,605,249	$7,059,958	$2,454,708	1.79	−29.25%

^a^Cost of PPMA = Total cost of PPMA include investment cost

^b^ *% Change ROI* (ROI new − ROI base)/(ROI base) * 100

When considering the public-pay perspective, the total cost of the PPMA program in 2014 was $342,464. Approximately 56.4% of the costs of the PPMA program were pharmacist fees for the service of prescribing for minor ailments, with the remaining 43.6% of costs being for government paid prescriptions. In comparison, when we adopt a societal perspective, the total cost of the PPMA program in 2014 was $655,947. In this scenario, societal costs of the PPMA program ($313,483) account for 47.7% of the total costs of the program. The societal costs comprised of training costs that accounted for 19.53% of total costs, prescription costs (i.e. private insurance or out-of-pocket payments), opportunity costs (i.e. wait and consultation time), and travel costs to the pharmacy of which accounted for 55.86, 23.94 and 0.67% respectively, of the total societal costs.

For the alternative scenario (absence of the PRMA program), we estimated that total costs for minor ailments from a public payer perspective for one year was $350,715. We found that 71.16% of the public-pay costs were associated with GP visits, 12.68% were from ER visits and 16.16% were attributable to government paid prescription costs. Finally, considering societal costs in the alternative scenario, we found that productivity losses from OTCs versus prescription medications accounted for the highest costs at 61.68%, with wait time for GP or ER, cost of OTC meds, travel costs and prescription costs accounting for 23.61, 6.50, 0.040 and 7.81%, respectively, of total patient costs (i.e. costs borne by patients).

## Discussion

Currently, there are major questions surrounding how health systems can address minor health conditions in more efficient and effective ways, including through the use of preventative medicine and by administering care in less expensive settings [40–43]. To address these questions, a variety of jurisdictions in other developed countries have attempted to develop functional alternatives to physician care, with many programs focusing on minor ailments. The resource constraints facing practically all health systems in the world led not only to the growing interest in cost-containment solutions that preserve quality of health care services but also to the need to evaluate such solutions. A major study was conducted to assess the cost-benefit of self-care initiatives in the EU and also assess the transferability of initiatives to different contexts [9]. The EU study evaluated the UK Minor Ailment Scheme (MAS) as one of the interventions representing self-care legal changes and found that this type of legislative change to be a favourable policy option from a societal perspective [9].

Studies show that alternatives to physician care (e.g. retail clinics, prescribing by allied professionals) generally have a similar level of quality to physician offices and ER visits, and are often more convenient and cheaper for patients [44–46]. Similarly, pharmacists prescribing programs have been lauded as one way to improve accessibility to primary care and prescription medicines. However, very few studies have looked at the costs and savings of running a pharmacist prescribing program, and how they could impact the efficiency of the health care system.

A study by the Ontario Pharmacists Association (OPA) determined that the implementation of a PPMA program aimed at ‘five key practice areas’, including “counselling and prescribing for smoking cessation, administering flu vaccinations, adapting patients’ drug therapy, renewing prescriptions for stable chronic conditions, and prescribing for minor ailments” could save the provincial health system $143 million over the next 5 years [47]. Moreover, one of the few published studies on costs and savings of pharmacists prescribing found that the pharmacists prescribing for minor ailments program in three primary care trusts (185 pharmacies) in North East England saved the local health authorities about £6739 per month [14]. The authors attributed the brunt of the cost-savings to patients using a cheaper alternative to traditional care (e.g. ER and GP visits). Another UK report estimated that cases of minor ailments seen at GP offices and emergency departments (ones that could be re-directed to community pharmacy) could consume approximately £1.1 billion in resources [3].

Our estimated savings from the public payer perspective were more conservative than those from Baqir et al. [14], likely because the remuneration paid to pharmacists was higher, a greater percentage of patients in the PPMA program said they would use self-care/OTCs rather than seeing a physician for their minor ailment and we included the cost of prescription medications in the analysis. The societal perspective did however find even greater savings from using a PPMA program than those found in the UK, this was largely due to the productivity loss associated with self-care over pharmacists prescribing. It is important that future cost analyses of PPMA programs carefully consider how they set up their analyses, specifically which of the most influential costs and savings to include (e.g. pharmacist remuneration, productivity loss, diversion of GP/ER visits) as they can make a major difference in the results of an analysis.

Previous research in Canada shows respondents would generally have a willingness to pay for this service, if it was not covered by the government or insurance, of approximately $18.95 per patient per consultation [48]. This is consistent with international research which indicates patients may be willing to pay pharmacists to prescribe, particular if it would mean shorter wait times [49, 50]. The Nova Scotia Report also noted that while many individuals would be willing to pay for the service, but that the cost would remain a barrier for many, particularly those of low income [48]. Our results demonstrate that it may be suitable from a cost-saving perspective for the government to cover the costs of running such a program, particular if we consider the results from the societal perspective.

There were some benefits of the PPMA we were not able to capture as part of our costing analysis, particularly the potential for improved accessibility to health care advice and a prescription medications. Studies show that many people with minor ailments often do not seek appropriate care [51–54]. One example of this was when pharmacists in British Columbia were first allowed to prescribe emergency contraception, which not only saved the province money but also increased access, with the number of emergency contraceptives provided increasing by over 100% [55]. The pilot study conducted on a PPMA program in Nova Scotia demonstrated that participants in the program felt the program aided in their access to assessment and prescriptions for minor ailments, and improved the timeliness of care [48].

The uptake of a PPMA program in different locations depends on a large number factors and seems to vary significantly. For instance, while Nova Scotia saw their pharmacists prescribing program had particular benefits in rural areas where there was limited access to GPs, a study by Wagner et al. [7] in Scotland indicated a lower uptake of the minor ailment pharmacists prescribing program in rural areas. Consequently, future research on the PPMA program in Canada should consider the rural uptake of the program and potential barriers to its use. The same Scottish study did find higher uptake of the minor ailment program in the most deprived areas (based on the 2006 Scottish index of multiple deprivation), suggesting pharmacists prescribing programs may be particularly beneficial to those most at risk of health problems and those with limited access to health care [7]. Therefore, although it is difficult to capture it in this costing analysis, the PPMA may also have the additional benefit of reaching an unmet need for minor ailments.

There are also potential limitations to the PPMA program not captured in this economic analysis. For instance, currently the program only compensates pharmacists when they prescribe a medication, therefore theoretically this program could encourage the unnecessary use of prescriptions. In effect, the pharmacist fees could create a potential conflict of interest for pharmacists who could earn money for both the prescribing and the dispensing of prescriptions [1, 56]. Another concern with pharmacists prescribing is whether the PPMA program can meet an appropriate standard of care, equivalent to a physician. However, evaluation research of these pharmacists prescribing programs, in Canada and in the UK, show high patient satisfaction and a high rate of symptom resolution [4, 5, 48]. Watson et al. [45] found that mean improvement in quality of life and symptom resolution was consistent between three different health care settings (pharmacists, GP offices and emergency departments). These findings justify our assumption that the health outcomes for the PPMA program and the alternative would be similar, and therefore to compare the two programs based on cost.

It is important to note some of the limitations of our analysis. For instance, we did not include the productivity loss due to lack of treatment for all the applicable minor ailments under the program, including acne, insect bites, oral aphthous ulcer, oral thrush, diaper dermatitis, atopic dermatitis, dysmenorrhea, hemorrhoids, skin infections and tinea skin infections. However, as these are not as commonly associated with productivity loss or were relatively rare conditions within this particular prescribing environment, we decided to stay conservative with our savings estimate. Furthermore, we were not able to measure the costs of continuing to run the PPMA program, as these estimates were not available to us. We also did not measure directly the cost of re-consultation rates (i.e. rate that a patient goes to see a doctor after consulting a pharmacist). However, the evidence indicates that re-consultations are very similar whether an individual originally consulted a pharmacist or a GP [7, 57] and that these re-consultation rates are generally quite low [4, 5]. Moreover, we did not include cost estimates of the impact of the side effects of the prescription medications, however the Saskatchewan Minor Conditions Survey estimated side effects were only an issue in 1.3% of cases.

This study investigated costs and savings associated with the PPMA program, which was the only feasible study design given the recent implementation of the program and data availability. However, future research should also focus on conducting full economic evaluations of PPMA programs to evaluate both the costs and benefits of having or not having these programs. Currently, legislation for prescribing by pharmacists exists in only a few countries and its expansion to a larger number of countries may partially depend on the economic evaluations of existing PPMA programs.

## Conclusion

As countries develop pharmacists prescribing programs with the aim of improving the efficiency of care, reducing pressure on the healthcare system, and increasing accessibility to prescription medications [1, 2], the need for evaluating these programs becomes more pressing. We conducted an economic analysis of the pharmacists prescribing for minor ailments PPMA program in Saskatchewan to estimate the costs and savings of the program from both the public payer and societal perspectives and also estimate the projected costs and savings of the program for a time horizon of five years after implementation. We found that the PPMA program in Saskatchewan was cost-saving from a public payer perspective and much more so from a societal perspective. Therefore, our results indicate that pharmacists prescribing programs have the potential to be cost-saving especially if savings to society are considered and may lead to improved access. While we think it is important to learn from other countries’ experiences, we also know that context matters in these types of evaluations and hence as other countries consider broadening pharmacists’ scope of practice, analysts should examine frameworks for policy transfer in health and conduct their own cost analysis and economic evaluations to account for significant differences in health care systems.

## Additional file

**Additional file 1.** List of prescribed drug for each minor ailment with their prices and markup. This table contains list of all prescribed drug for 14 minor ailment along with their price and mark-up fee in PPMA.

## Abbreviations

PPMA: pharmacists prescribing for minor ailments
ROI: return on investment
GERD: gastroesophageal reflux disease
CIHI: Canadian Institute for Health Information
OTC: over trade counter
EAC: equivalent annual cost
CADTH: Canadian Agency for Drugs and Technologies in Health
