# Supplementary material for: Costs and savings associated with a pharmacists prescribing for minor ailments program in Saskatchewan
Source: Cost Eff Resour Alloc. 2017 Apr 11;15:3. doi: 10.1186/s12962-017-0066-7 (PMC5387257; doi:10.1186/s12962-017-0066-7)
Supplement: Supplementary file 1 — Additional file 1. List of prescribed drug for each minor ailment with their prices and markup. This table contains list of all prescribed drug for 14 minor ailment along with their price and mark-up fee in PPMA. [file 12962_2017_66_MOESM1_ESM.doc]

**Additional file [1] List of prescribed drug for each minor ailment with their prices and markup**

| **Ailment** | **Prescription drug** | **Price** | **Markup*** |
| --- | --- | --- | --- |
| Acne | Benzaclin Gel Pump | 51.76 | 5.18 |
|  | Clindoxyl Gel | 44.63 | 4.46 |
|  | Retin-A crm 0.05% | 13 | 1.95 |
|  | Stieva-A crm | 14.76 | 2.21 |
|  | Differin 0.1% gel/crm | 121.3 | 12.13 |
|  | Benzaclin Gel Pump | 51.76 | 5.18 |
| **Average prescription drug price for Acne** (54.28$)** | | **49.1$** | **5.2$** |
| Allergic Rhinitis | Mometasone Nasal Sp 50mcg | 23.1 | 2.31 |
|  | Fluticasone Nasal Sp 50mcg | 23.4 | 2.34 |
|  | Omnaris Nasal Sp 50mcg | 27.97 | 2.80 |
| **Average prescription drug price for Allergic Rhinitis (27.31$)** | | **24.8$** | **2.5$** |
| Atopic Dermatitis | Mometasone furoate 0.1% crm | 8.41 | 1.26 |
|  | Hydroval 0.2% oint | 2.67 | 0.80 |
|  | Betamethasone 0.05% crm | 8.52 | 1.28 |
| **Average prescription drug price for Atopic Dermatitis (7.65$)** | | **6.53** | **1.11** |
| Cold sores | Vatrex 500mg | 29.89 | 2.99 |
|  | Valacyclovir 500mg | 7.34 | 1.10 |
| **Average prescription drug price for Cold sores (20.66$)** | | **18.6$** | **2.04$** |
| Diaper Dermatitis | Ketoderm 2% crm | 10.1 | 1.5 |
| **Average prescription drug price for Diaper Dermatitis (11.64$)** | | **11.64$** | **1.5$** |
| Dysmenorrhea | Mefenamic acid 250mg | 12.75 | 1.91 |
|  | Naproxen 500mg | 6.74 | 1.01 |
| **Average prescription drug price for Dysmenorrhea (11.21$)** | | **9.7$** | **1.4$** |
| GERD | Omeprazole 20mg | 13.16 | 1.97 |
|  | Tecta 40mg | 24.41 | 2.44 |
|  | Pantoprazole 40mg | 11.59 | 1.74 |
|  | Esomeprazole 40mg | 59.72 | 5.97 |
| **Average prescription drug price for GERD (30.2$)** | | **27.2$** | **3.03$** |
| Headache | Ibuprofen 600mg | 1.96 | 0.59 |
|  | Naproxen 500mg | 3.15 | 0.95 |
|  | Rizatriptan ODT 10mg | 23.68 | 2.37 |
|  | Sumatriptan 100mg | 50.23 | 5.02 |
| **Average prescription drug price for Headache (21.9$)** | | **19.7$** | **2.2$** |
| Hemorrhoids | Anusol-HC oint | 24.88 | 2.49 |
|  | Anugesic-HC oint | 29.62 | 2.96 |
|  | Proctosedyl oint | 27.02 | 2.70 |
|  | Proctol oint | 19.6 | 1.96 |
|  | Proctol suppositories | 9.8 | 1.47 |
|  | Anugesic-HC suppositories | 17.77 | 1.78 |
|  | Anusol-HC suppositories | 26.06 | 2.61 |
| **Average prescription drug price for Hemorrhoids (24.3$)** | | **22.1$** | **2.2$** |
| Strains & Sprains | Ibuprofen 600mg | 1.96 | 0.20 |
|  | Naproxen 500mg | 3.15 | 0.95 |
|  | Celecoxib 200mg | 5.3 | 1.59 |
| **Average prescription drug price for Strains & Sprains (4.38$)** | | **3.4$** | **0.9$** |
| Canker Sores | Oracort | 8.6 | 1.2 |
| **Average prescription drug price for Canker Sores (9.92$)** | | **8.6$** | **1.2$** |
| Oral Thrush | Nystatin suspension | 6.7 | 1.01 |
| **Average prescription drug price for Oral Thrush( 7.71$)** | | **6.7$** | **1.01$** |
| Skin Infections | Fucidin 2% oint | 23.53 | 2.35 |
|  | Mupirocin 2% oint | 5.52 | 1.66 |
| **Average prescription drug price for Skin Infections ( 16.5$)** | | **14.5$** | **2$** |
| Tinea Corporis Skin | Ketoderm 2% crm | 10.12 | 1.52 |
|  | Lamisil 1% crm | 17.12 | 1.71 |
| **Average prescription drug price for Tinea Corporis Skin ( 15.2$)** | | **13.6$** | **1.6$** |
| **Average Prescription drug for minor ailments** ( 18.80$)** | | **16.8$** | **2.01$** |
| **Dispending Fee** | | **11.40$** | |
| **Average Prescription drug for minor ailments including Dispending fee** | | **30.20$** | |
| **Minimum Prescription drug for minor ailment + Dispending fee ( $21.22)** | | **$8.52** | **$1.30** |
| **Maximum Prescription drug for minor ailment + Dispending fee ( $43.94)** | | **$29.3** | **$3.18** |

*Markup fee calculated based on percentage of drug price which varies between 10%-30% as following: 30% for drug cost up to $6.30; 15% for drug cost between $6.31 and $15.80; 10% for drug cost of $15.81 to $200.00, and a maximum mark-up of $20.00 for drug cost over $200.00.

**Average prescription drug price for each ailments = Average price of prescription drug + Average markup
